# The Extracellular Domain of Two-component System Sensor Kinase VanS from Streptomyces coelicolor Binds Vancomycin at a Newly Identified Binding Site

**DOI:** 10.1038/s41598-020-62557-z

**Published:** 2020-03-31

**Authors:** Christine Lockey, Richard J. Edwards, David I. Roper, Ann M. Dixon

**Affiliations:** 1 MOAC Doctoral Training Centre, University of Warwick, Coventry, CV4 7AL UK; 2 Medical Research Council Doctoral Training Centre, University of Warwick, Coventry, CV4 7AL UK; 3 School of Life Sciences, University of Warwick, Coventry, CV4 7AL UK; 4 Department of Chemistry, University of Warwick, Coventry, CV4 7AL UK

**Keywords:** Biochemistry, Biochemistry, NMR spectroscopy, Biophysics, Biological fluorescence, Membrane biophysics, Antimicrobial responses

## Abstract

The glycopeptide antibiotic vancomycin has been widely used to treat infections of Gram-positive bacteria including *Clostridium difficile* and methicillin-resistant *Staphylococcus aureus*. However, since its introduction, high level vancomycin resistance has emerged. The genes responsible require the action of the two-component regulatory system VanSR to induce expression of resistance genes. The mechanism of detection of vancomycin by this two-component system has yet to be elucidated. Diverging evidence in the literature supports activation models in which the VanS protein binds either vancomycin, or Lipid II, to induce resistance. Here we investigated the interaction between vancomycin and VanS from *Streptomyces coelicolor* (VanS_SC_), a model Actinomycete. We demonstrate a direct interaction between vancomycin and purified VanS_SC_, and traced these interactions to the extracellular region of the protein, which we reveal adopts a predominantly α-helical conformation. The VanS_SC_-binding epitope within vancomycin was mapped to the N-terminus of the peptide chain, distinct from the binding site for Lipid II. In targeting a separate site on vancomycin, the effective VanS ligand concentration includes both free and lipid-bound molecules, facilitating VanS activation. This is the first molecular description of the VanS binding site within vancomycin, and could direct engineering of future therapeutics.

## Introduction

Vancomycin is a glycopeptide antibiotic that exerts its antimicrobial effect on a variety of Gram-positive bacterial species, and has been seen as a last line of defence in the treatment of complicated clinical infections including *Clostridium difficile* and methicillin-resistant *Staphylococcus aureus* (MRSA). Despite the significant toxicity of vancomycin in patients, there is still strong clinical reliance on this drug as a last line of defence against such infections^1^. In some cases, MRSA infections are incalcitrant and vancomycin-resistant Enterococcal (VRE) strains are resistant to a wide panel of antibiotics. As a result, VRE and MRSA strains are now on worldwide health authorities’ high priority lists, and new treatments are desperately needed^1–3^. Three recently approved semi-synthetic glycopeptide derivatives, oritavancin, dalbavancin and telavancin, are being treated as reserve antibiotics to be used sparingly to preserve their efficacy^4^.

The mode of action of vancomycin involves binding to the d-alanyl-d-alanine-terminating pentapeptide of the cell wall precursor Lipid II, preventing its utilisation in biosynthesis of bacterial peptidoglycan cell wall^5^. Vancomycin is one of a number of antibiotics that target Lipid II rather than the enzymes that use Lipid II as a substrate^6^. Inducible high-level resistance to vancomycin is commonly found in soil bacteria including the Actinomycetes^7^ where it has been genome-encoded for millennia^8^. Recently resistance genes have spread intergenerically into *S. aureus*, resulting in the first strains of vancomycin- and methicillin-resistant *S. aureus* (VRSA) causing widespread clinical concern^9^. High-level vancomycin resistance is induced through the upregulation of genes in the transposon-encoded *van* gene cluster and was first characterised in a VRE isolate^5,10–12^. These genes encode a series of enzymes that coordinate to break down the cell’s supply of d-alanyl-d-alanine for peptidoglycan biosynthesis, and replace it with d-alanyl-d-lactate^13,14^. The resulting d-alanyl-d-lactate-containing Lipid II is then integrated into the cell wall by existing cellular machinery^15^; the affinity of this modified precursor for vancomycin is reduced approximately 1000-fold, and vancomycin is therefore unable to disrupt the synthesis of the bacterial cell wall peptidoglycan, resulting in vancomycin-resistant cell growth^5,10^.

**Figure 1:**
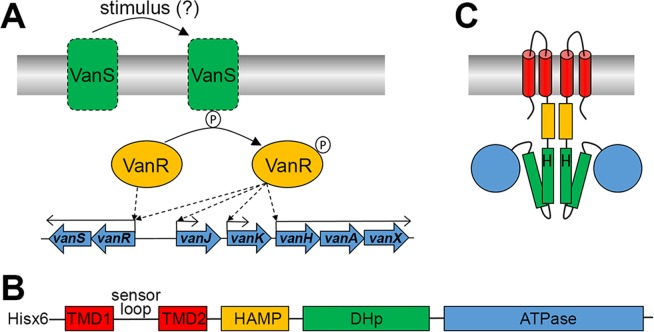
(**A**) The regulatory role of VanSR on *vanHAX* transcription in Actinomycetes. VanS is stimulated, in the presence of vancomycin, to undergo autophosphorylation and subsequent phosphotransfer to VanR. Compared to VanR, Phospho-VanR has an increased binding affinity for the promoter regions of *vanR* as well as *vanH*, *vanJ* and *vanK*. In the absence of vancomycin, VanR remains dephosphorylated, and binds only to the promoter region of *vanR* to maintain low-level expression of VanSR. (**B**) Predicted architecture of VanS from *S. coelicolor* (VanS_SC_) based on homology modelling. The extracellular sensor loop domain is flanked by two transmembrane domains (TMD 1–2), followed by an intracellular HAMP domain, a dimerisation and histidine phosphorylation (DHp) domain which is the site of autophosphorylation via a conserved histidine, and an ATPase domain. Location of the amino terminal hexahistidine tag on the protein described in this study is also indicated. (**C**) Predicted topology of the domains in VanS_SC_.

The upregulation of genes in the *van* cluster is controlled by the VanSR two-component regulatory system (Fig. 1A)^7,16^. VanS is an integral membrane protein and sensor histidine kinase, with a domain architecture and predicted topology as shown in Fig. 1B-C^17^, and is essential for the detection and response to the presence of vancomycin in the cellular environment. In the presence of such a stimulus, VanS is thought to dimerise, undergo autophosphorylation in its cytoplasmic kinase domain, and then transfer the acquired phosphate group to VanR, the cytosolic response regulator^18^. Phosphorylated VanR binds upstream of the *van* transcription elements, and induces expression of these proteins^19^.

While the role of VanS in inducing vancomycin resistance is well characterised and understood, the exact mechanism by which VanS initially detects vancomycin in the cellular environment remains the subject of debate. Evidence in the literature supports different models in which VanS is activated by direct binding of vancomycin^20–23^, or by accumulation of uncrosslinked Lipid II (or vancomycin-Lipid II complex), a by-product of vancomycin activity^24,25^. A thorough characterisation of the VanS activation mechanism is required to understand this biological mechanism and may provide the knowledge base to resensitise vancomycin-resistant bacteria to continued use of this vital antibiotic.

*Streptomyces coelicolor* is a model Actinomycete species which displays inducible vancomycin resistance of the VanB phenotype. The VanB phenotype is one of six recognised resistance phenotypes (VanA–E, VanG) of vancomycin-resistant bacteria, all of which involve the alteration of Lipid II. VanA and VanB strains are highly resistant to vancomycin making them the most clinically relevant phenotypes, and have a highly similar domain architecture (see Figure S1), but they are differentiated from one another by their resistance to the antibiotic Teicoplanin: VanA strains are resistant to Teicoplanin; VanB strains are sensitive. The VanS protein in *S. Coelicolor*, VanS_SC_, is a 42 kDa integral membrane protein^17^ and the subject of the work presented here. This protein is of particular interest following the report by Koteva and co-workers^22^ that it acts as a vancomycin receptor. Specifically, it was suggested that a putative vancomycin-binding site is located at the N-terminus of VanS_SC_ between residues 1–41, which includes the putative first transmembrane helix and residues D_38_-W_41_ of the sensor loop. However, to our knowledge, no further refinement of this location nor any molecular model of this interaction site has been proposed since, and there is no X-ray or NMR structure of the protein available. We wished to define the molecular determinants for vancomycin recognition in this system, both from within vancomycin itself as well as within VanS.

In this report, we demonstrate the direct interaction between vancomycin and purified VanS_SC_ using a range of biochemical and biophysical methods including solution state nuclear magnetic resonance (NMR) spectroscopy. We have studied both the full-length VanS_SC_ protein and a synthetic peptide corresponding to its extracellular domain in order to trace these interactions to the extracellular “sensor” region of the protein, a region that we also show has a highly α-helical secondary structure. Utilising the protons in vancomycin as chemical probes of interaction, we have used NMR to map the VanS binding site within vancomycin to a region at the N-terminus of the peptide chain that is almost entirely distinct from the lipid II binding site through which vancomycin’s antimicrobial activity is achieved. To our knowledge, this is the first molecular description of the VanS binding site within vancomycin, and could direct design of new therapeutics or modification of current antibiotics.

## RESULTS

### Purified full-length VanS_SC_ is well-folded, dimeric and active in detergent micelles

**Figure 2:**
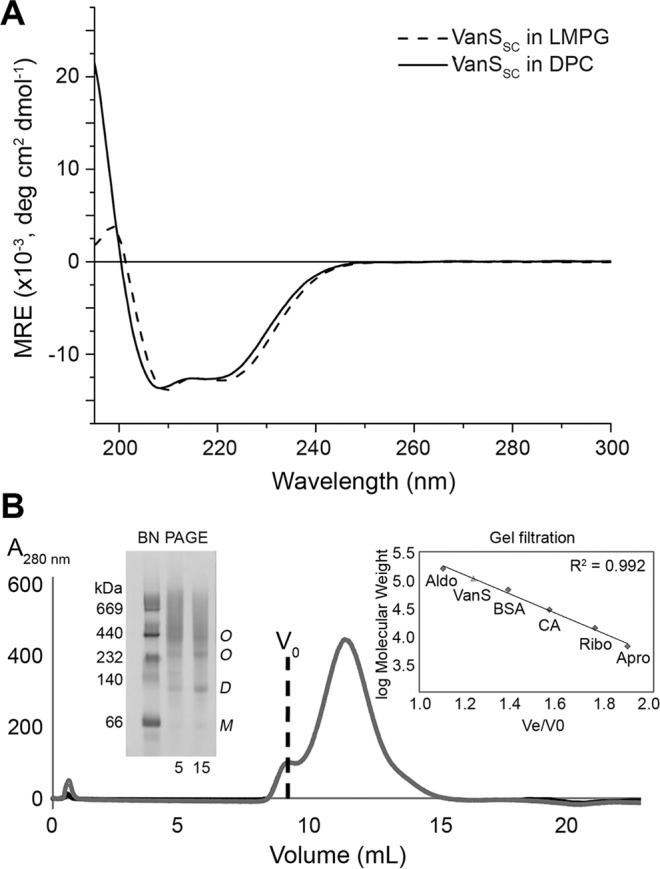
(**A**) Circular dichroism spectra acquired of 0.1 mg/mL His_6_-VanS_SC_ in 10 mM sodium phosphate buffer pH 6.8, 25 mM NaCl, and 2 mM DPC or 1.15 mM LMPG, showing the characteristic features of an α-helical protein with peaks at 208 and 222 nm. (**B**) Gel Filtration Chromatogram for sample of VanS_SC_ applied to a Superose 12 column (separation range 1–300 kDa). An isocratic gradient of 100% B was used (**B**: 20 mM HEPES pH 7.8, 300 mM NaCl, 0.075% w/v DPC). Inset (left hand side) shows BN-PAGE 4–16% gradient gel loaded with purified His-tagged full-length VanS_SC_ (5 μg and 15 μg) solubilised in DPC detergent at 0.1% w/v (~2x CMC). An estimate of protein molecular weight was provided by a NativePAGE protein marker (MR, Amersham). Bands at molecular sizes corresponding to monomer (*M*), dimer (*D*) or higher oligomer (*O*) are indicated. Inset (right hand side) shows a calibration curve of gel filtration standards. A plot of elution volume (Ve)/void volume (V_0_) versus log molecular weight is shown for each of five standards (diamonds): Aldolase (‘Aldo’, 158 kDa), Bovine Serum Albumin (‘BSA’, 66.9 kDa), Carbonic Anhydrase (‘CA’, 29 kDa), Ribonuclease A (‘Ribo’, 13.7 kDa) and Aprotinin (‘Apro’, 6.5 kDa). The result for VanS_SC_ is also shown on this plot (triangle).

Full-length VanS_SC_ from *S. coelicolor*, containing an N-terminal His_6_ tag, was expressed in *E. coli* and purified as detailed in the Methods. Solubilisation of the protein in the zwitterionic detergent dodecylphosphocholine (DPC) and the lysolipid 1-myristoyl-2-hydroxy-sn-glycero-3-phospho-(1′-rac-glycerol) (LMPG), both detergents with an excellent track record of solubilising membrane proteins for structural studies^26–29^, yielded highly similar CD spectra that suggested high α-helical content from the negative peaks at 208 and 222 nm **(**Fig. 2A). The maximum helical content was calculated from mean residue ellipticity at 222 nm^30^ to be approximately 32%. In the absence of a three-dimensional structure for VanS_SC_, the Sequence Annotated by Structure (SAS) tool of EMBL-EBI^31^ was used to predict the percentage helicity of VanS_SC_ from its amino acid sequence. The tool estimated that VanS_SC_ is 36% α-helical, in good agreement with our results, suggesting that the protein is similarly well-folded in DPC and LMPG micelles.

A further test of how effectively micelles preserve the fold of VanS_SC_ is the retention of the native oligomeric state of the protein. Although the oligomeric state of VanS_SC_ has yet to be reported, sequence analysis indicates the presence of a HAMP domain, implicated in signalling of specifically dimeric sensor kinases^21,32^. We investigated the oligomeric state of DPC-solubilised VanS_SC_ using gel filtration chromatography (GFC) and blue native PAGE (BN-PAGE). Apart from protein aggregates (>300 kDa) which eluted in the void volume (V_0_ ~ 9.0 mL), VanS_SC_ analysed by GFC eluted in a single peak centred at an elution volume (Ve) of 11.5 mL (Fig. 2B). Comparison of the Ve to those obtained from gel filtration standards (Fig. 2B (inset right) and S2) suggests that the primary species for VanS_SC_ in DPC is that of a dimer (~80–90 kDa, monomeric molecular weight = 42.6 kDa), reflecting the predicted native oligomeric state of this protein. This propensity to oligomerise was also observed by BN-PAGE, which provides a measure of molecular size relative to standards in non-denaturing conditions^33^; DPC supported formation of discrete VanS_SC_ oligomers (Fig. 2B inset left, lanes 2 and 3). We interpret the weak band just below 66 kDa as monomeric VanS_SC_, and note that although this is ~50% above the theoretical mass of the protein, proteins with pI values ≥ 9.3 typically display slow migration in BN-PAGE reflecting inaccurately high molecular mass^19,33^. In a standard SDS-PAGE analysis, our purified protein migrated in accordance with its theoretical mass (Figure S2, inset). Given that our N-terminally His_6_ tagged VanS_SC_ protein has a pI = 9.4, we assigned the lowest band in the BN-PAGE gel to a monomeric species with confidence. A more pronounced dimer band was visible on the Coomassie-stained gel (*D*), suggesting that this protein forms stable dimers in detergent micelles. Two higher molecular weight bands (at approximately 230 and 440 kDa, both labelled *O* for oligomer) were also visible, and may represent higher-order association of VanS_SC_ dimers.

**Figure 3:**
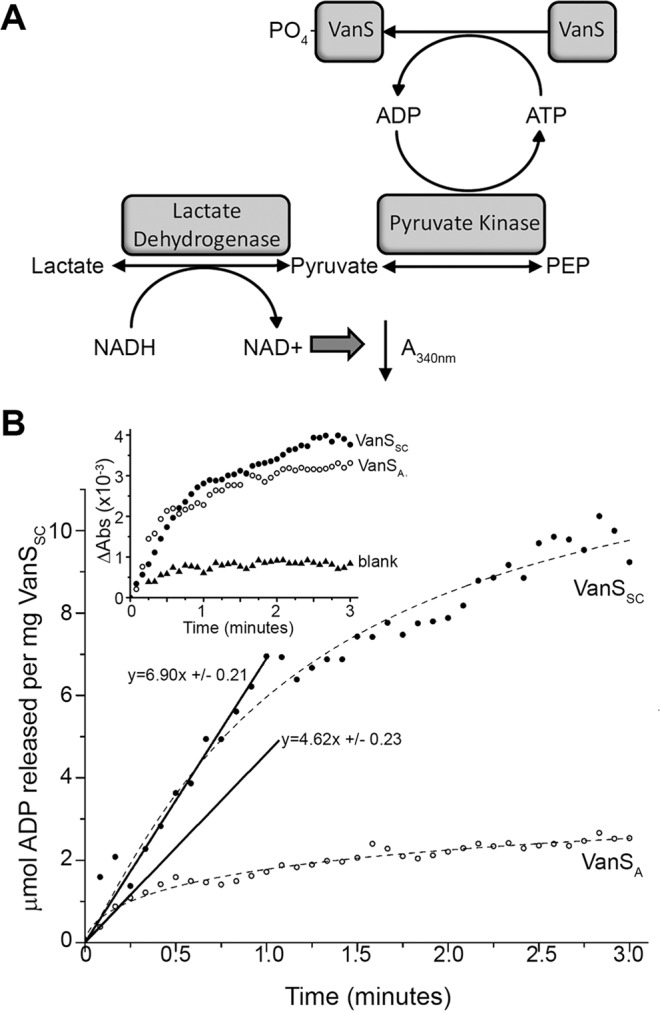
(**A**) The coupled ADP-release assay used to assess constitutive autophosphorylation activity of His_6_-VanS_SC_. Following autophosphorylation, released ADP is regenerated to ATP by pyruvate kinase, and phosphoenol pyruvate (PEP) is converted to pyruvate. Lactate dehydrogenase converts pyruvate to lactate, oxidising NADH to NAD+ . The loss of NADH in the sample is measured as a reduction in absorbance at 340 nm, which can therefore be utilised as an indirect measure of ADP release by VanS_SC_. (**B**) Turnover of ADP by His_6_-VanS_SC_ (13.7 μM) over the course of a 3-minute continuous photometric kinase activity assay. μmol ADP released is estimated from the change in absorbance of the solution at 340 nm, caused by the conversion of NADH to NAD+ . The specific activity of His_6_-VanS_SC_ in 1.15 mM LMPG was estimated to be 6.9 μmol ADP released per mg kinase per minute. The activity of His_6_-VanS_A_ was also assayed as a positive control (34.1 μM; see Supplementary Information), and estimated to be 4.6 μmol ADP released per mg kinase per minute.

Although the helical content and dimer formation mirrored the *in vivo* properties of VanS_SC_, the ability of this protein to function as a kinase or phosphatase may be affected when solubilised in detergents. Previous studies have shown that the addition of detergent can hamper or prevent detection of the autophosphorylation and dephosphorylation rate of histidine kinases^34^. Therefore, it was critical to assess the activity of purified VanS_SC_ in a detergent environment prior to further study. We elected to use a continuous assay for ADP production^35^, to measure the autophosphorylation activity of purified VanS_SC_ in a detergent micelle context (Fig. 3A). This assay is based upon continuous turnover of ATP upon autophosphorylation^36^, facilitating quantification of an autophosphorylation rate.

LMPG is well-suited to these activity measurements due to the low critical micelle concentration (CMC) of this detergent (0.2 mM^37^), which minimises any impact on the activities of the coupling enzymes in the assay (*i.e*. PK, LDH) by excess detergent molecules. Conversely, DPC has a 7-fold higher CMC (1.5 mM^37^) which can lead to denaturation of soluble enzymes. Because these detergents produced similar protein folds as assessed using CD, activity measurements were only performed in LMPG. The initial rate of reaction was obtained from the plot of ADP release versus time (Fig. 3B), and used to calculate a specific activity for His_6_-VanS_SC_ of 6.90 ± 0.21 µmol mg^−1^ min^−1^. To ensure that a contaminating ATPase was not responsible for this activity, turnover of ADP was also measured in the absence of any VanS_SC_ (Fig. 3B inset), and these values were subtracted from all data. This activity was compared directly to that of an A-type VanS from *Enterococcus faecium* (VanS_A_) purified and solubilised in the same manner (see Supplementary Information), which yielded a specific activity of 4.62 ± 0.23 µmol mg^−1^ min^−1^. VanS_A_ was used as a positive control since its activity has been demonstrated *in vitro* in detergent micelles and amphipols and found to autophosphorylate in all cases^23,38^. The specific activities of VanS_SC_ and VanS_A_ are very similar as are the kinetics of autophosphorylation, with both approaching maximum autophosphorylation after three minutes (Fig. 3B). The activities observed are also in good agreement with values reported in the literature for other kinases^35^, which range from 1–59 µmol mg^−1^ min^−1^, suggesting that VanS_SC_ activity was maintained in detergent micelles and the protein was suitable for further biophysical study.

### Fluorescence data yield evidence of direct binding between full-length VanS_SC_ and Vancomycin

**Figure 4:**
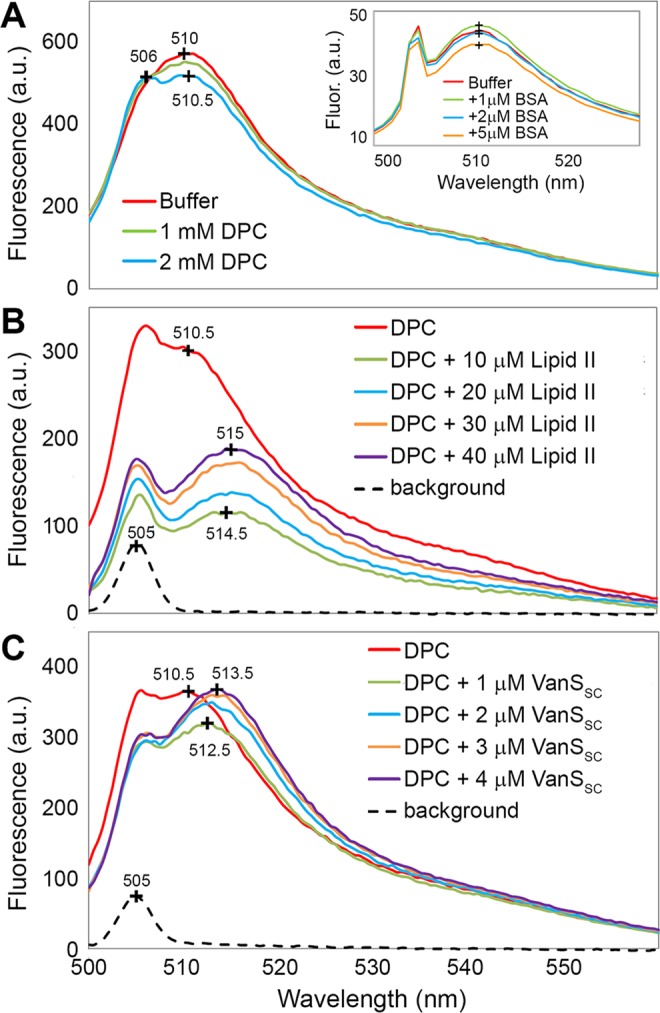
Fluorescence emission spectra, following excitation at 505 nm, of BODIPY-vancomycin (10 μM) dissolved in (**A**) 20 mM HEPES pH 7.8 in the absence and presence of 1–2 mM DPC detergent, (*inset*) 20 mM HEPES pH 7.8 containing 1–5 μM of our negative control bovine serum albumin (BSA), (**B**) 20 mM HEPES pH 7.8 containing 2 mM DPC and 10–40 µM Lipid II, and (**C**) 20 mM HEPES pH 7.8 containing 2 mM DPC and 1–4 µM VanS_SC_. (**B**,**C**) also show “background” fluorescence emission spectra collected in the absence of BODIPY-vancomycin for solutions of 10 µM Lipid II and 1 µM VanS_SC_, respectively. Both spectra were acquired in 2 mM DPC-containing HEPES buffer and are shown as dashed lines.

The direct binding of a fluorescent BODIPY-vancomycin analogue (Figure S3) to purified, detergent solubilised VanS_SC_ was investigated using fluorescence spectroscopy, and used to report upon any interaction between full-length VanS_SC_ and the antibiotic. Although BODIPY-vancomycin has a small Stokes shift, with an excitation wavelength (λ_ex_) of 504 nm and an emission wavelength (λ_em_) of 510 nm, the high fluorescence quantum yield and sharp, solvent-insensitive emission peaks make this probe well-suited for ligand binding studies in detergent environments. The λ_em_ and intensity of BODIPY-vancomycin were very similar in the presence and absence of DPC detergent micelles (Fig. 4A), confirming the low environmental dependence of the BODIPY fluorophore. A second peak at ~506 nm was present in all detergent-containing samples, and likely results from light scattering in the detergent solution.

The magnitude of the change in BODIPY λ_em_ under the conditions used here was first evaluated using Lipid II, which is known to bind strongly to vancomycin with a binding constant of 54 μM^10,39,40^. Titration of Lipid II into DPC-solubilised BODIPY-vancomycin yielded a red shift in λ_em_ of 4.5 nm (Fig. 4B), indicating that Lipid II and BODIPY-vancomycin interact as expected. This interaction was nearly saturated at the lowest Lipid II concentration studied (10 μM), with successive additions of Lipid II having little impact on λ_em_. This reflects the reported 1:1 stoichiometry of Lipid II:Vancomycin binding^39^. This approach was then applied to study of BODIPY-vancomycin interactions with VanS_SC_. Addition of 1 μM VanS_SC_ yielded a red shift in λ_em_ of 2 nm (Fig. 4C), and further aliquots of VanS_SC_ were added until no appreciable change in the spectrum was observed. Emission spectra of the BODIPY dye did not display the same level of quenching in these experiments, but a cumulative red shift in λ_em_ of 3 nm was observed, which is similar to that of the Lipid II positive control, and suggests that full-length VanS_SC_ interacts directly with the vancomycin analogue. To confirm the specificity of this interaction, the experiment was repeated substituting bovine serum albumin (BSA) as a putative binding partner for BODIPY-vancomycin, given that BSA has a large number of readily accessible α-helices. Addition of BSA from 1 μM to 5 μM yielded no shift in λ_em_ of BODIPY-vancomycin (Fig. 4A, inset), indicating that the red shift observed in the presence of VanS_SC_ represents a selective interaction between VanS_SC_ and the antibiotic.

### VanS_SC_ sensor-loop region forms an α-helix in the presence of a membrane mimetic and experiences significant change in dynamics upon addition of vancomycin

The fluorescence results above lead to the obvious question of where the vancomycin-binding site was located within our purified VanS_SC_. Full-length VanS_SC_ was subjected to two- and three-dimensional heteronuclear NMR analyses but produced broad NMR signals (data not shown) due to the slow tumbling of the detergent-solubilised dimer in solution (analogous to a > 100 kDa protein). As a result we focused our attention on a region of the extracellular portion of VanS_SC_ which may provide an interaction site with ligands like vancomycin. Given that the extracellular “sensor” domain shown in Fig. 1 is the most exposed and accessible region for ligand binding, a synthetic peptide containing VanS_SC_ residues D38-F62, predicted to lie between TMD 1 and 2 and thus comprise the extracellular loop, was synthesised and purified. Topology analysis of the VanS_SC_ sequence to yield the sequence of the extracellular region, here termed “sensor peptide”, is detailed in Methods. The membrane mimetic used in NMR studies was 100 mM DPC-d_38_/10 mM DPPC-d_75_ mixed micelles (or bicelles), as it has been reported that addition of 1,2-dipalmitoyl-sn-glycero-3-phosphocholine (DPPC) to detergents improves the quality of NMR data while providing a more native-like membrane environment, without a loss in signal-to-noise^41^.

**Figure 5:**
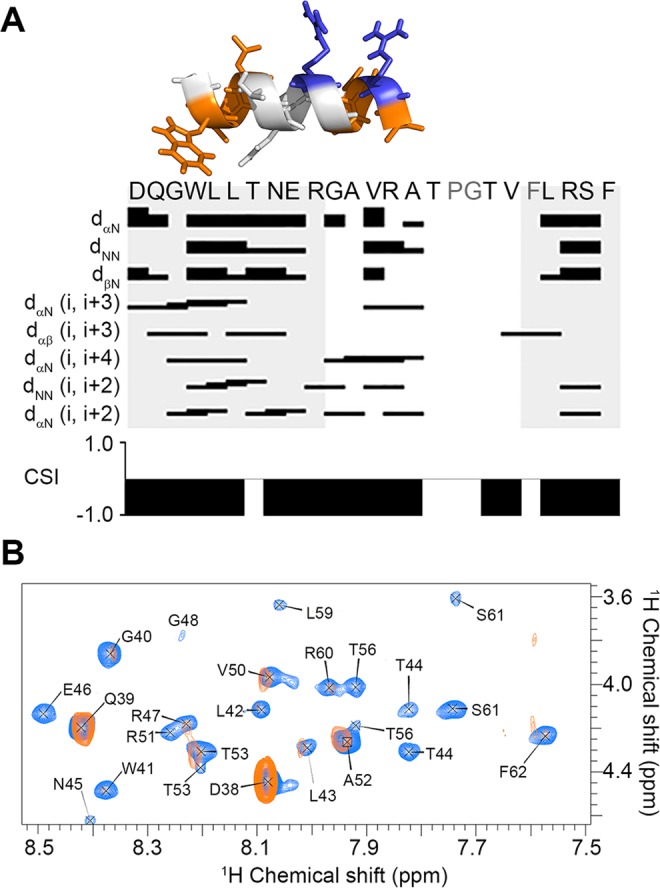
(**A**) Survey of NMR-derived sequential backbone NOE connectivities for the VanS_SC_ sensor peptide (D38-F62) solubilised in DPC-d_38_/DPPC-d_75_ mixed micelles (25 mM sodium phosphate, pH 6.8), classified as strong, weak, or absent by the thickness (or absence) of a bar connecting the residues involved. The non-sequential connectivities listed (*i.e*. i, i + n) are unique to α-helices, and were used alongside chemical shift index analyses (CSI) to localise α-helical stretches yielding a CSI of -1. Also shown is a molecular model of the continuous helical stretch from G40-A52, with basic residues coloured blue and hydrophobic residues coloured orange, indicating an amphipathic nature to the α-helix. (**B**) The assigned H_N_ - Hα fingerprint region of equivalent TOCSY spectra acquired for 1 mM sensor peptide in DPC/DPPC mixed micelles in the absence (blue) and presence (orange) of 1 mM vancomycin HCl. The H_N_ - Hα crosspeaks of some residues (*e.g*. Q_39_) are unaffected, while those of others (*e.g*. T44) are fully attenuated.

2-dimensional homonuclear NMR experiments, namely total correlation spectroscopy (TOCSY^42,43^) and nuclear Overhauser enhancement spectroscopy (NOESY^43^) experiments, were used to sequentially assign the sensor peptide in the presence of DPC-d_38_/DPPC-d_71_ mixed micelles (Figure S4, Table S1). Assignment was possible for all but three residues (P54, G55 and F58). Chemical shift index analysis^44,45^ of the ^1^Hα chemical shifts for each residue indicates that the peptide is at least 72% α-helical (Fig. 5A). Long-range backbone (i, i + n) H_N_, Hα and Hβ NOEs indicative of α-helix formation were detected in the presence of mixed micelles (Fig. 5A) and suggested a continuous helical stretch between residues G40-A52. A molecular model of this region is shown in Fig. 5A, with basic Arg residues shown in blue and hydrophobic residues shown in grey. The arrangement of these residues on discrete helical faces is similar to that observed in cationic amphipathic helices (APHs), known to have a natural affinity for bacterial membranes. To our knowledge, this is the first report of any regular structure for the sensor region of any VanS protein.

In the presence of a 1:1 molar ratio of vancomycin HCl, a considerable loss of crosspeak intensity was observed for protons in the sensor peptide in both the TOCSY and NOESY experiments (Fig. 5B). This attenuation of peaks precluded use of chemical shift to probe binding interactions, as severe broadening reduced the ability to measure chemical shift accurately. However, loss of crosspeak intensity itself can be indicative of ligand binding, with increased linewidths resulting from changes in dynamics or chemical shift averaging for nuclei in the binding site. With this in mind, TOCSY data were used to compare the relative loss of crosspeak intensity along the length of the sensor peptide. NOESY data were excluded from analyses since interpretation of NOESY crosspeak intensity is complicated by changes in internuclear distance, cross-relaxation and exchange rates in the free and bound species^46^.

Figure 5B shows the significant attenuation of crosspeaks resulting from scalar coupling of the backbone amide and alpha protons (NH-Hα) in TOCSY spectra collected in the absence (blue) and presence (orange) of vancomycin using identical acquisition and processing parameters. All but two residues (D_38_ and Q_39_) that yielded observable crosspeaks in this region experienced severe attenuation in crosspeak volume upon addition of vancomycin. Such widespread broadening of peaks could be an indication of protein aggregation, leading to increased broadening and eventual disappearance of all peaks. However, no visible change in the samples was observed (*e.g*. precipitation or cloudiness) and attenuation was much less pronounced in other regions of the NMR spectrum (Figure S5). For example, cross peaks in the aromatic and aliphatic regions of the spectrum were retained and did not show any pronounced change in chemical shift (as would be expected from large-scale aggregation events).

To investigate whether vancomycin could have this same effect on any highly helical peptide on the surface of a membrane, we acquired an equivalent dataset from a sample of vancomycin and the model amphipathic helix composed of residues 2–23 of human α-synuclein^47^ in DPC-d_38_/DPPC-d_75_ mixed micelles. We have previously demonstrated this peptide is highly helical in bicelles and detergent micelles^48^. Here we observed no chemical shift change or attenuation of α-synuclein TOCSY crosspeaks (Figure S6) upon addition of an equimolar concentration of vancomycin, indicating that the signal intensity loss observed upon addition of vancomycin is selective for the VanS_SC_ sensor peptide.

### Vancomycin binds to VanS_SC_ sensor-loop peptide via a region distinct from the Lipid II D-Alanyl-D-Alanine binding site

An orthogonal ligand-directed approach was also taken to monitor the nuclei within vancomycin under the same experimental conditions in order to locate the VanS_SC_-binding site within vancomycin. To our knowledge, no molecular model of this interaction site within the antibiotic has been proposed before, nor is it known whether this site maps to the Lipid II-binding site or is separate from that site. Therefore, we wished to reveal a molecular description of this binding site with atomic resolution using solution-state NMR spectroscopy.

**Figure 6:**
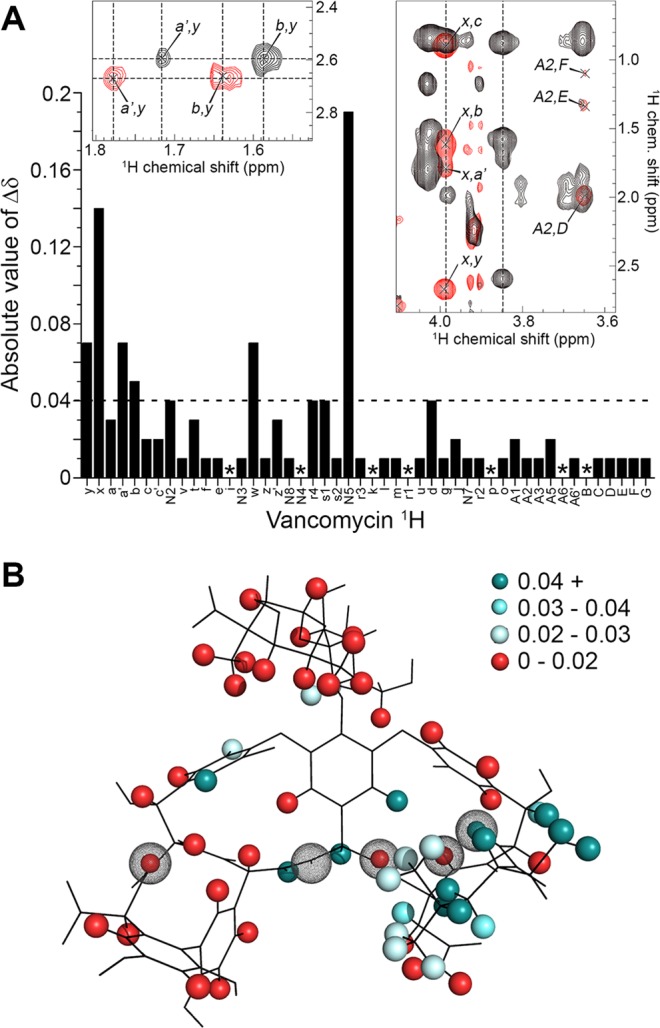
(**A**) The change in chemical shift (Δδ) of each proton in vancomycin HCl upon addition of an equimolar quantity of the VanS_SC_ sensor loop peptide. Asterisks indicate protons for which no Δδ is observed. Insets show overlaid spectra before (black) and after (red) addition of sensor peptide to highlight resolution of peaks that shift by ≥ 0.04 ppm Δδ. (**B**) The crystal structure of vancomycin (**PDB ID: 1FVM**, crystallised in complex with the lipid II pentapeptide terminus) onto which protons observable in acquired NMR spectra are mapped as solid spheres. The signal perturbations experienced by all observable protons in the presence of the sensor peptide are shown as a heat map on the structure above, with protons experiencing the largest signal perturbation (Δδ ≥ 0.04 ppm, 3 × standard error) shown in dark cyan, and then varying in shade between Δδ = 0.04–0.02 ppm. Protons experiencing Δδ from 0–0.2 ppm are shown in red to represent negligible perturbation. Atoms involved in hydrogen bonding to lipid II are indicated by dotted spherical cage representations.

Using ^1^H-^1^H TOCSY and ^1^H-^1^H NOESY spectra, the ^1^H resonances of vancomycin were assigned in three separate conditions: aqueous buffer; mixed micelles; and sensor peptide solubilised in mixed micelles. In 25 mM sodium phosphate buffer, pH 6.8, 96% of the assignable ^1^Hs in vancomycin were readily identified and the assignments were consistent with published values for vancomycin in CD_3_COOD-NaOD-D_2_O, pD 5.5 at 318 K^49^ and in (CD_3_)_2_SO, pD 6.0 at 298 K^50^ (Figure S7). Vancomycin was then re-assigned in mixed micelles in the absence (Table S2, Figure S8) and presence of a 1:1 molar ratio of sensor peptide, and the chemical shift changes are shown in Fig. 6A. We note that these chemical shift changes are small, but readily observable in the data (Fig. 6A, insets). A chemical shift change (Δδ) equal to 0.04 ppm represents three standard errors above the average. Protons that experienced significant chemical shift changes in the presence of the sensor peptide are mapped as a heat map according to the magnitude of Δδ onto the vancomycin structure in Fig. 6B.

Inspection of Fig. 6B revealed a discrete cluster of protons within or near the Leu residue at the N-terminus of the peptide component of vancomycin which were selectively impacted by the presence of the sensor peptide. We suggest that this region of vancomycin is involved in binding to the sensor peptide. To confirm the specificity of this interaction, the experiment was repeated using the membrane-bound amphipathic helix, α-synuclein, as a negative control in place of the VanS_SC_ sensor loop. In this case, very few vancomycin protons experienced chemical shift perturbation (see Figure S9), and these protons do not cluster together to form a recognisable binding site. Also shown in Fig. 6B are the hydrogen and oxygen atoms which form the d-Alanyl-d-Alanine binding site upon binding of vancomycin to Lipid II^39^. These atoms are indicated by spherical “cages” to identify their location, and are involved in key hydrogen bonding interactions that stabilise the vancomycin-Lipid II complex. Interestingly, only one proton (N2) is shared between those affected by the sensor peptide and those known to interact with Lipid II, suggesting that the sensor peptide-binding site is distinct from the Lipid II binding site.

Several protons that lie outside of the putative sensor peptide “binding site” also respond to the addition of the sensor peptide. Vancomycin is known to form both dimers and higher-order oligomers in some circumstances^51^ and specifically, has been reported to form dimers at concentrations above 1.4 mM^52^. Although vancomycin was added here at a concentration below 1.4 mM (*i.e*. 1 mM), we cannot rule out the formation of vancomycin dimers under these conditions. The vancomycin protons that experience significant chemical shift changes and lie at the dimer interface in the vancomycin dimer structure (**PDB ID: 1AA5**) are shown in Figure S10 (denoted by spherical cages). One of these protons (N5, see Figure S8) yielded the largest chemical shift change observed in this study.

## Discussion

VanS is a large, homodimeric integral membrane protein. As such, structural characterisation of VanS is highly challenging. While recent studies have demonstrated production of highly pure and active samples of A-type VanS^38,53,54^ which have been characterised using techniques including circular dichroism and analytical ultracentrifugation, characterisation via high-resolution methods such as X-ray crystallography and NMR spectroscopy has met with very little success. Thus far, structural understanding of VanS has been gleaned from comparison to other sensor histidine kinases of known structure. However, while the transmembrane and cytosolic components of sensor histidine kinases show significant homology, this class of protein is known to respond to a wide variety of stimuli^55^, and this is reflected in the variety of solved structures of sensor histidine kinase extracellular domains in the literature^56–58^. This lack of structural characterisation of the VanS extracellular domain has hampered our understanding of the architecture of this protein and any ability to target VanS therapeutically. Here we describe characterisation of the secondary structure content and oligomeric state of a purified and active B-type VanS from *S. coelicolor*, VanS_SC_. We show that the predominant oligomeric state of the protein is that of a dimer, reflecting the assembly of this protein *in vivo*. The secondary structure content also reflects that of other histidine kinase family members. The dimeric form of purified VanS_SC_ we observe in ligand-free protein preparations is in contrast to the predominantly monomeric form of the related A-type VanS observed in the absence of ligand by Phillips-Jones and co-workers^54^. This difference may be due to differences in the solution conditions used; VanS oligomerisation was measured here in detergent micelles and was measured previously in a detergent-free solution supplemented with 20% glycerol^54^.

Our results agree well, however, with the results for A-type VanS mentioned above in that our data demonstrate direct vancomycin-VanS_SC_ interactions *in vitro* using a purified and active VanS_SC_ sample. Previously a highly functionalised vancomycin analogue was proposed to interact directly with VanS_SC_. Koteva *et al*.^22^ reported binding of VanS_SC_ to a vancomycin photoaffinity probe in *E. coli* membrane fractions. The work we present here is unique in that binding has been demonstrated in a purified sample of VanS_SC_ with chemically unchanged vancomycin, supporting the hypothesis that a direct interaction between vancomycin and VanS is a means of signalling in the antibiotic resistance mechanism. We note that the link between vancomycin binding and VanS activity is still the subject of much debate, with separate recent studies of the A-type VanS from *E. faecium* reporting that enzyme activity is both sensitive to^23^ and insensitive to^38^ vancomycin *in vitro*.

The sensor peptide used in this work, derived from the extracellular region of VanS_SC_, readily folds into a highly α-helical structure in the presence of membrane mimetics. This is the first report, to our knowledge, of any higher order structure in this region of VanS_SC_. Given that this extracellular region, anchored by flanking transmembrane domains, must always be in close proximity to a membrane surface we suggest that the high propensity for helical structure we observe here may represent the native fold. High helical propensities in the extracellular loops of other integral membrane proteins including G-protein-coupled receptors^59,60^, transporters^61^, Na/Ca exchangers^62^, tetraspanins^63^, and the cell division protein FtsX^64^ have been reported previously. The presence of these helical elements is often thought to direct specific interactions with binding partners. For example, in the case of Fe(III)-phytosiderophore transporters, the helical propensity of the seventh extracellular loop is thought to control substrate specificity^61^. Likewise, the second extracellular loop of the tetraspanin human occludin has been shown to adopt a helical fold, and the authors link this fold to the unique ability of this region to interact with other endothelial tight junction proteins^61^. These and other studies give weight to the argument that the helical structure we observe in the VanS_SC_ extracellular loop is not an artefact, but instead reflects formation of a structured binding site for vancomycin (and possibly other antibiotics). We also note that the arrangement of basic Arg residues and strongly hydrophobic residues on discrete helical faces in this region of VanS_SC_ is similar to that observed in cationic APHs, known to have a natural affinity for bacterial membranes which are enriched in anionic lipids. Such an interaction is commonly observed for antimicrobial peptides, which use their cationic nature to selectively target and lyse bacterial membranes^65^. It is plausible that an interaction with the bacterial membrane could facilitate the prearrangement of a binding site; indeed, this has been shown recently for a bacterial glycosyltransferase which utilises cationic helix-bacterial membrane interactions to direct productive binding between the jaw domain and Lipid II^66^. Thorough analysis of peak volumes in all TOCSY spectra revealed that significant broadening of main chain ^1^H NMR signals occurred along almost the entire length of the sensor region upon addition of a 1:1 molar ratio of vancomycin, precluding identification of an isolated binding site in the protein, but strongly suggestive of a large-scale change in mobility. Taken together, our data indicate binding of the antibiotic vancomycin to a highly helical VanS_SC_ extracellular domain.

We have mapped the VanS_SC_ sensor domain-binding site in vancomycin to the N-terminal leucine of the polypeptide chain within the antibiotic, and find that the VanS binding site is distinct from the D-alanyl-D-alanine binding site on Lipid II. Such a distinction may provide a system for detection of vancomycin in both Lipid II-bound and unbound states; thus, for VanS_SC_ to target a distinct binding site not obscured by vancomycin-lipid II binding is evolutionarily advantageous. It is worth noting that deletion of this N-terminal leucine in vancomycin inhibits antibiotic activity^67^, so VanS_SC_ binding cannot easily be evaded by mutation of the sidechain. However, a predictable consequence of this separate binding site is the emergence of modified glycopeptides to which VanS_SC_ cannot bind, but which are capable of binding to lipid II. Indeed, this has already been reported^17^. To our knowledge, a secondary protein-binding site on any glycopeptide antibiotic has not been reported until now. This understanding could guide design and screening of therapeutic molecules that block activation of the two-component system. Our data also support a model in which sensor peptide binding may stimulate vancomycin dimerisation. Such behaviour would facilitate binding to the sensor region of an adjacent VanS_SC_ chain (which would be present in the VanS_SC_ dimer). Dimerisation of glycopeptide antibiotics has previously been suggested to offer a binding advantage over monomers in solution, facilitating interactions between antibiotics and their binding targets by inducing accumulation of the antibiotic at the membrane surface^68^.

## Methods

### Heterologous overexpression and purification of full-length His_6_-VanS_SC_

The sequence encoding the *S. coelicolor* VanS histidine kinase (UniProt ID Q9X942_STRCO) was codon optimised for *E. coli* and cloned into the pProEX vector containing an N-terminal hexahistidine tag to create the pProEx::His_6_-VanS_SC_ plasmid. *E. coli* C41 (DE3) pRIL transformed with pProEx::His_6_-VanS_SC_ was cultured at 37 °C, 180 rpm, in Luria-Burtani medium containing 35 μg/mL chloramphenicol and 100 μg/mL carbenicillin. At OD_600_ = 0.6, expression of VanS_SC_ was induced by the addition of 200 μM isopropyl β-d-1-thiogalactoside (IPTG). Cultures were then incubated at 25 °C, 180 rpm, overnight and cells were harvested by centrifugation at 3,000 × g at 4 °C for 30 minutes. Cells were homogenised in 25 mM sodium phosphate buffer at pH 6.8, containing 25 mM NaCl and 10% glycerol, by two passes through a Constant Systems cell disruptor at 25 kpsi, 4 °C. Cell debris was removed from lysates by centrifugation at 20,000 × g at 4 °C for 30 minutes. Membranes were then pelleted by centrifugation at 200,000 × g for 2 hours at 4 °C, and resuspended in binding buffer (25 mM sodium phosphate buffer at pH 6.8, containing 25 mM NaCl and 10 mM dodecylphosphocholine (DPC) or 6.7 mM 1-myristoyl-2-hydroxy-sn-glycero-3-phospho-(1′-rac-glycerol) (LMPG) (Avanti Polar Lipids, Alabaster, USA). Purification was achieved using immobilised metal affinity chromatography (IMAC) by cycling clarified, detergent-solubilised membranes through a 5 mL prepacked HisTrap HP column (GE Life sciences, USA) at a rate of 1 mL/min for 2 hours at 4 °C, followed by washing of the column in binding buffer, binding buffer with 1 M NaCl, and binding buffer with 50 mM imidazole, and subsequent eluting of His_6_-VanS_SC_ in binding buffer with 300 mM imidazole. The purity of all protein fractions at each stage of preparation was assessed using sodium dodecyl sulphate polyacrylamide gel electrophoresis (SDS-PAGE, visualised using both Coomassie Blue R250 and silver nitrate) and immunoblotting against the His_6_ tag.

### Analytical gel filtration chromatography

Oligomerisation of full-length VanS_SC_ solubilised in DPC detergent micelles was analysed using analytical gel filtration chromatography on a 10/300 mm Superose 12 column (GE Healthcare) attached to an AKTA Prime system (GE Healthcare). Equilibration of the column was performed with Buffer A (Buffer A: 20 mM HEPES, pH 7.8, 200 mM NaCl, and 0.075% DPC) before protein samples were loaded. Separation was accomplished at a flow rate of 0.3 mL/min under isocratic conditions until a volume corresponding to 1.5 column bed volumes (36 mL) was collected in 0.3 ml fractions. Protein absorbance was recorded at 280 nm and 254 nm wavelengths, and the elution volume (Ve) of VanS_SC_ was determined and compared to those obtained for a set of protein standards (GE Healthcare) under identical conditions to yield an estimation of molecular weight. The standards used were as follows: were aprotinin (‘Apro’, 6.5 kDa); ribonuclease A (‘Ribo’, 13.7 kDa); carbonic anhydrase (‘CA’, 29 kDa); bovine serum albumin (‘BSA’, 66.9 kDa); and aldolase (‘Aldo’, 158 kDa).

### Blue native-PAGE

Oligomerisation of full-length VanS_SC_ solubilised in DPC detergent micelles was also analysed using the NativePAGE Novex Bis-Tris system (Invitrogen), based on the BN-PAGE technique^33^. Proteins were separated on a 4–16% Bis-Tris gradient gel at 4 °C in NativePAGE running buffer (1 M Bis-Tris-HCl pH 7.0, 1 M Tricine pH 7.0). Samples were prepared in a non-denaturing Coomassie G-250 dye, which confers a net negative charge while maintaining the proteins in their non-denatured native state. Proteins were visualised using Coomassie Brilliant Blue and molecular weights were estimated relative to prestained markers (High MW Standards, Amersham).

### Autophosphorylation activity assay

The native activity of full length VanS_SC_ was measured by a coupled enzymatic activity assay^35^. 13.7 μM His_6_-VanS_SC_ was incubated at room temperature with 1 mM ATP, 6 units PK, 2 mM PEP, 8.4 units LDH, and 100 μM NADH, in the presence of 50 mM KCl and 1 mM DTT in 25 mM sodium phosphate buffer pH 6.8, 25 mM NaCl, 1.15 mM LMPG. The phosphorylation assay could not be performed in DPC; presumably monomeric DPC, present at 1–1.5 mM concentrations due to its relatively high CMC, adversely affects the activity of one of the enzymes in the assay.

### Fluorescence spectroscopy

The interaction between vancomycin and full-length VanS_SC_ was initially measured by fluorescence spectroscopy. A 10 μM solution of fluorescently labelled vancomycin probe, BODIPY-vancomycin (Molecular Probes, Sigma-Aldrich), in 20 mM HEPES buffer pH 7.8, was excited at 504 nm on a Perkin Elmer LS50 fluorometer with slit width 1 nm and scan speed 50 nm/min at 20 degrees, and its emission spectrum monitored from 500 to 600 nm. To investigate the impact of micelles on BODIPY-vancomycin fluorescence, equivalent spectra were recorded from the sample following addition of DPC to a concentration of 1 mM and 2 mM. Subsequently, to investigate the impact of a known vancomycin ligand on fluorescence, spectra were recorded following addition of Lipid II to the sample of BODIPY-vancomycin and 2 mM DPC, to concentrations of 10, 20, 30, and finally 40 μM. A stock solution of 660 μM Lipid II was prepared in 2:3:1 chloroform:methanol:water, and aliquots of this stock were dried under nitrogen. Successive Lipid II films were then solubilised into a sample of BODIPY-vancomycin, DPC and HEPES buffer, to yield mixed BODIPY-vancomycin/Lipid II samples for the investigation. Finally, to investigate binding between vancomycin and VanS_SC_, samples of BODIPY-vancomycin, 2 mM DPC, and 1, 2, 3 and 4 μM VanS_SC_ were generated and emission spectra recorded.

### Peptide synthesis and purification

The transmembrane domains of VanS_SC_ were predicted via analysis of the sequence of the VanS histidine kinase from *S. coelicolor* (UniProt ID Q9X942_STRCO) using Phoibus. A 25-residue peptide corresponding to the extracellular loop in VanS_SC_ (residues 38–62), with the sequence DQGWLLTNERGAVRATPGTVFLRSF, was synthesised using F-moc chemistry and purified to 95% purity at Insight Biotechnology Limited (Wembley, UK). Similarly, a peptide containing residues 2–23 of human α-synuclein, with the sequence DVFMKGLSKAKEGVVAAAEKTK, was also synthesised. Peptide purity was confirmed by HPLC and matrix-assisted time of flight mass spectrometry (MALDI-TOF-MS, Bruker) before subsequent lyophilisation. The peptide was stored as dry powder at −20 °C until use.

### Circular dichroism spectroscopy

CD spectra were collected on Jasco J-815 or J-720 spectropolarimeters (Jasco UK, Great Dunmow, UK) equipped with Peltier temperature control and xenon light sources. VanS_SC_ samples contained 0.42 mg/mL (10 μM) protein, 2 mM DPC or 1.15 mM LMPG, 25 mM sodium phosphate (pH 6.8), and 25 mM NaCl. Peptide samples contained 0.5 mg/mL (100 μM) VanS_SC_ sensor peptide, 100 mM DPC or LMPG, 25 mM sodium phosphate (pH 6.8), and 25 mM NaCl. Spectra were recorded between 190 nm and 280 nm, with a bandwidth of 2 nm and a data pitch of 0.2 nm. The CD spectrum of the buffer was recorded as a blank and was subtracted from each protein spectrum. The percentage of secondary structure content was estimated from mean residue ellipticity at 222 nm^30^.

### NMR experiments

All NMR experiments were performed in 3 mm NMR tubes (Bruker, Germany) on an Avance 700 MHz spectrometer (Bruker Biospin, UK) equipped with a triple resonance inverse cryoprobe with Z-gradients. Data were processed using Topspin 3.0 (Bruker Biospin), and analysed using Topspin 3.0 and CcpNmr Analysis (University of Leicester, UK).

Peptide samples for NMR analyses were prepared by dissolving the peptide to a final concentration of 0.8–1 mM in 25 mM Na_2_HPO_4_ (pH 6.8), 90% H_2_O, 10% D_2_O, containing 100 mM DPC-d_38_ and 10 mM DPPC-d_75_^1^H-^1^H TOCSY and NOESY NMR spectra were recorded with 4096 × 256 data points or 4096 × 512 data points at a temperature of 25 °C. TOCSY and NOESY mixing times ranged from 70–140 ms and 80–180 ms, respectively. Chemical shift indices for peptide α-protons were calculated^45^ using random coil reference values^44^. Percent attenuation of peptide crosspeak volumes collected for samples containing no vancomycin (V_free_) or a 1:1 molar ratio of vancomycin:peptide (V_Bd_) were calculated according to the following equation: % Attenuation = (V_free_ - V_Bd_/V_free_) × 100.

## Supplementary information

Supplementary information.
