# Supplementary material for: The Extracellular Domain of Two-component System Sensor Kinase VanS from Streptomyces coelicolor Binds Vancomycin at a Newly Identified Binding Site
Source: Sci Rep. 2020 Mar 31;10:5727. doi: 10.1038/s41598-020-62557-z (PMC7109055; doi:10.1038/s41598-020-62557-z)
Supplement: Supplementary file 1 — Supplementary information. [file 41598_2020_62557_MOESM1_ESM.docx]

# SUPPLEMENTARY INFORMATION FOR:

***The Extracellular Domain of Two-component System
Sensor Kinase VanS from Streptomyces coelicolor***

***Binds Vancomycin at a Newly Identified Binding Site***

Christine Lockey^a^, Richard J. Edwards^b^, David I. Roper^c^, and Ann M. Dixon^d*^

^a^MOAC Doctoral Training Centre,

^b^Medical Research Council Doctoral Training Centre,

^c^School of Life Sciences, and

^d^Department of Chemistry, University of Warwick, Coventry, CV4 7AL, UK.

*To whom correspondence should be addressed: Dr Ann Dixon, Department of Chemistry, University of Warwick, Coventry, CV4 7AL, UK, Telephone: +44 2476 150037; FAX: +44 2476 524112; email: [ann.dixon@warwick.ac.uk](mailto:ann.dixon@warwick.ac.uk)

**Running Title:** Vancomycin binding to VanS from *S. coelicolor*

***Heterologous Overexpression and Purification of full-length His_6_-VanS_A_***

The sequence encoding the VanS histidine kinase from *E. faecium* (UniProt ID VANS_ENTFC) was codon optimised for *E. coli* and cloned into the pProEX vector containing an N-terminal hexahistidine tag to create the pProEx::His_6_-VanS_A_ plasmid. *E. coli* C41 (DE3) pRIL transformed with pProEx::His_6_-VanS_A_ was cultured at 37°C, 180 rpm, in Luria-Burtani medium supplemented with 100 μg/mL carbenicillin and 35 μg/mL chloramphenicol. At OD_600_ = 0.6, expression of VanS_A_ was induced by the addition of 200 μM isopropyl β-d-1-thiogalactoside (IPTG). Cultures were then incubated at 25°C, 180 rpm, overnight and harvested by centrifugation at 3,000 × g for 30 minutes at 4°C. Cells were homogenised in 25 mM sodium phosphate pH 6.8, 25 mM NaCl, 10% glycerol by two passes through a Constant Systems cell disruptor at 25 kpsi, 4°C. Cell debris was removed from lysates by centrifugation at 20,000 × g for 30 minutes at 4°C. Membranes were then pelleted by centrifugation at 200,000 × g for 2 hours at 4°C, and resuspended in binding buffer (25 mM sodium phosphate pH 6.8, 25 mM NaCl, and 10 mM dodecylphosphocholine (DPC) or 6.7 mM 1-myristoyl-2-hydroxy-sn-glycero-3-phospho-(1'-rac-glycerol) (LMPG) (Avanti Polar Lipids, Alabaster, USA). Purification was performed using immobilised metal affinity chromatography (IMAC) by cycling clarified, detergent-solubilised membranes through a 5 mL prepacked HisTrap HP column (GE Life sciences, USA) at a rate of 1 mL/min for 2 hours at 4°C, followed by washing of the column in binding buffer, binding buffer with 1 M NaCl, and binding buffer with 50 mM imidazole, and subsequent eluting of His_6_-VanS_A_ in binding buffer with 300 mM imidazole. The purity of all protein fractions at each stage of preparation was assessed using sodium dodecyl sulphate polyacrylamide gel electrophoresis (SDS-PAGE, visualised using both Coomassie Blue R250 and silver nitrate) and immunoblotting against the His_6_ tag.

***VanS_A_ Autophosphorylation Activity Assay***

The native activity of full length VanS_A_ was measured by a coupled enzymatic activity assay [[36](#_ENREF_36)]. 34.1 μM His_6_-VanS_A_ was incubated at room temperature with 1 mM ATP, 6 units PK, 2 mM PEP, 8.4 units LDH, and 100 μM NADH, in the presence of 50 mM KCl and 1 mM DTT in 25 mM sodium phosphate buffer pH 6.8, 25 mM NaCl, 1.15 mM LMPG. The phosphorylation assay could not be performed in DPC; presumably monomeric DPC, present at 1-1.5 mM concentrations due to its relatively high CMC, adversely affects the activity of one of the enzymes in the assay.

CLUSTAL O(1.2.4) multiple sequence alignment

**VanSA:** SP|Q06240|VANS_ENTFC

**VanSSC:** TR|Q9X942|Q9X942_STRCO

SP|Q06240|VANS_ENTFC --------MVIKLKNKKNDYSKLERKLYMYIVAIVVVAIVFVLYIRSMIRGKLGDWILSI 52

TR|Q9X942|Q9X942_STRCO MDRRPGLSVRLKLTLSYAGFLTLAGVLLLVAV------GVFLL---------DQGWLLTN 45

: :**. . .: .* * : * **:* .*:*:

SP|Q06240|VANS_ENTFC LENKYDLNHLDAMKLYQYSIRNN---IDIFIYVAIVISILILCRVMLSKFAKYFDEINTG 109

TR|Q9X942|Q9X942_STRCO ERGAVRA---TPGTVFLRSFAPTAAWVMAFLLVFGLVGGWFLAGRMLAPLDRITEATRTA 102

.. .:: *: . : *: * ::. :*. **: : : : .*.

SP|Q06240|VANS_ENTFC IDVLIQNEDKQIELSAEMDVMEQKLNTLKRTLEKREQDAKLAEQRKNDVVMYLAHDIKTP 169

TR|Q9X942|Q9X942_STRCO ATG---SLSHRIRLPGRRDEYRELADAFDEMLAR--LEAHVAEQRR--FAANASHELRTP 155

. .::*.* .. * .: :::.. * : :*::****: .. :*:::**

SP|Q06240|VANS_ENTFC LTSIIGYLSLLDEAPDMPVDQKAKYVHITLDKAYRLEQLIDEFFEITRYNLQTITLTKTH 229

TR|Q9X942|Q9X942_STRCO LAVSKAILDVARTDPHQDPGEIIDRLHAVNTRAI---DLTEALLLLSRAGQ--RSFTREQ 210

*: . *.: *. .: . :* . :* :* : :: ::* . ::*: :

SP|Q06240|VANS_ENTFC IDLYYMLVQMTDEFYPQLSAHGKQAVIHAPEDLTVSGDPDKLARVFNNILKNAAAYS--- 286

TR|Q9X942|Q9X942_STRCO VDMSLLAEEATETLLPFAEKHGVTLETRGHVT-LALGSPALLLQLTTNLVHNAIVHNLPG 269

:*: : : *: : * . ** :. . *.* * :: .*:::** .:.

SP|Q06240|VANS_ENTFC EDNSIIDITAGLSGDVVSIEFKNTGSIP-KDKLAAIFEKFYRLDNARSSDTGGAGLGLAI 345

TR|Q9X942|Q9X942_STRCO RGRVWIHTAAGP--RTTRLVVENTGDLISPHQASTLTEPFQRGTERIHTDHPGVGLGLAI 327

... *. :** .. : .:***.: .: ::: * * * : :* *.******

SP|Q06240|VANS_ENTFC AKEIIVQHGGQIYAESND-NYTTFRVELPAMPDLVDKRRS 384

TR|Q9X942|Q9X942_STRCO VNTITQAHDGTLTLTPRHSGGLRVTVELPAAAPHTGR--- 364

.: * *.* : .. . . ***** ..:

**Figure S1:** Sequence alignment for VanA and VanB-type VanS proteins featured in this work, highlighting the highly similar domain architecture for these proteins despite the differences in their sensitivity to Teicoplanin. The positions of the various domains are highlighted in different colors, with transmembrane domains shown in yellow, HAMP domains shown in green, and histidine kinase domains shown in blue.

**
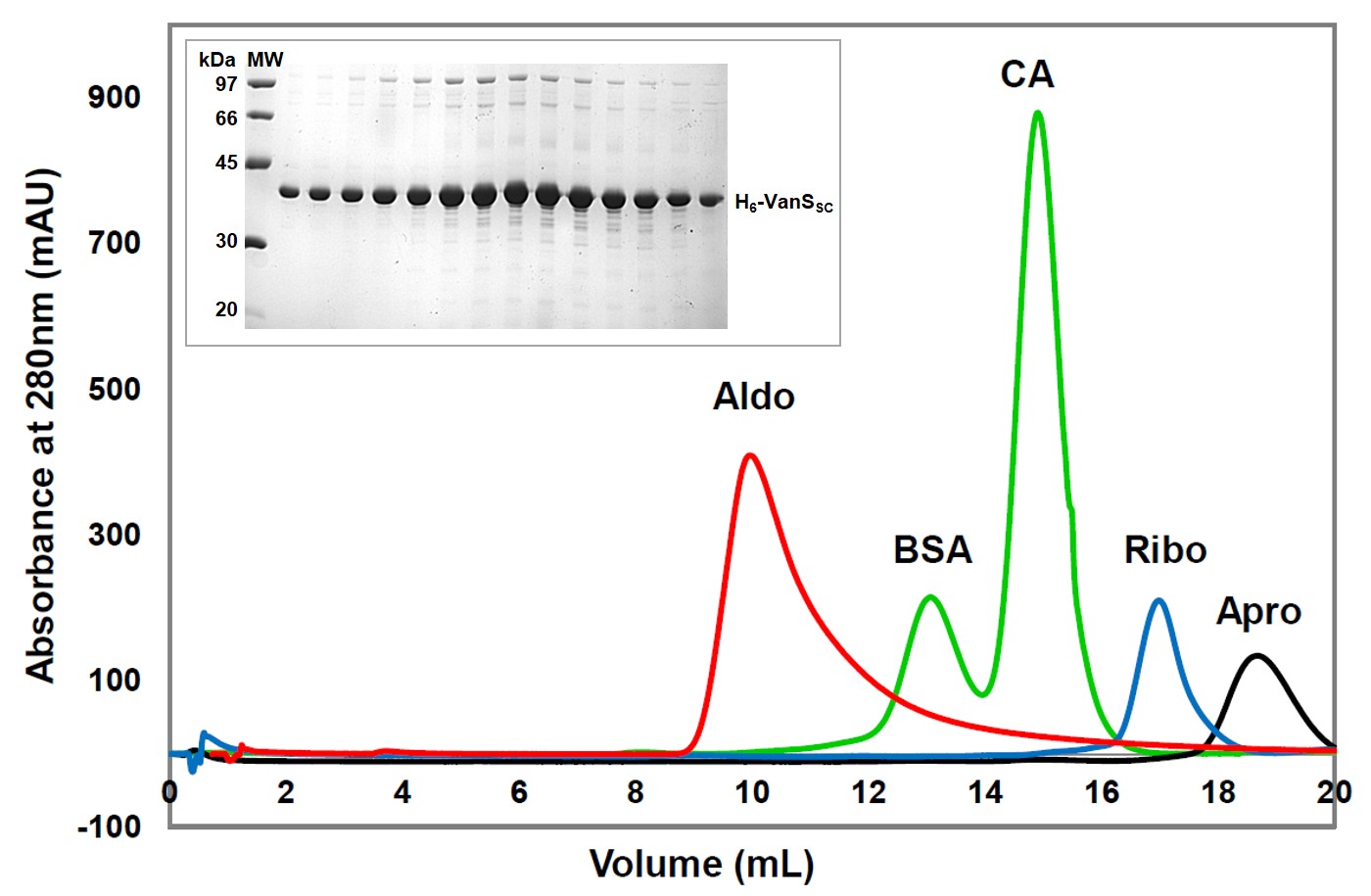
**

**Figure S2.** Gel filtration chromatograms of GE Healthcare standards applied to a Superose 12 10/300GL column, under an isocratic gradient of 100% B. Buffer B: 20 mM HEPES pH 7.8, 300 mM NaCl, 0.075% w/v DPC. Standards were: Aldolase (‘Aldo’, 158 kDa), Bovine Serum Albumin (‘BSA’, 66.9 kDa), Carbonic Anhydrase (‘CA’, 29 kDa), Ribonuclease A (‘Ribo’, 13.7 kDa) and Aprotinin (‘Apro’, 6.5 kDa). CA and BSA samples were added in the same sample, all other standards were run separately. Inset, A 12% SDS-PAGE gel showing aliquots of 0.3 mL gel filtration elutions collected under the main peak (~10 -12 mL) shown in Figure 2B.

Figure S3. Molecular structure of the fluorescent probe BODIPY-vancomycin (Sigma Aldrich). The BODIPY tag (red) is bound to the vancosamine sugar of the vancomycin molecule (black).

**Figure S4.** Assigned fingerprint region of 140 ms TOCSY spectrum acquired of 1 mM VSC_EC-25_ in 100 mM DPC-d_38_, 10 mM DPPC-d_71_, 25 mM sodium phosphate buffer pH 6.8, 25 mM NaCl, 10% D_2_O.

**
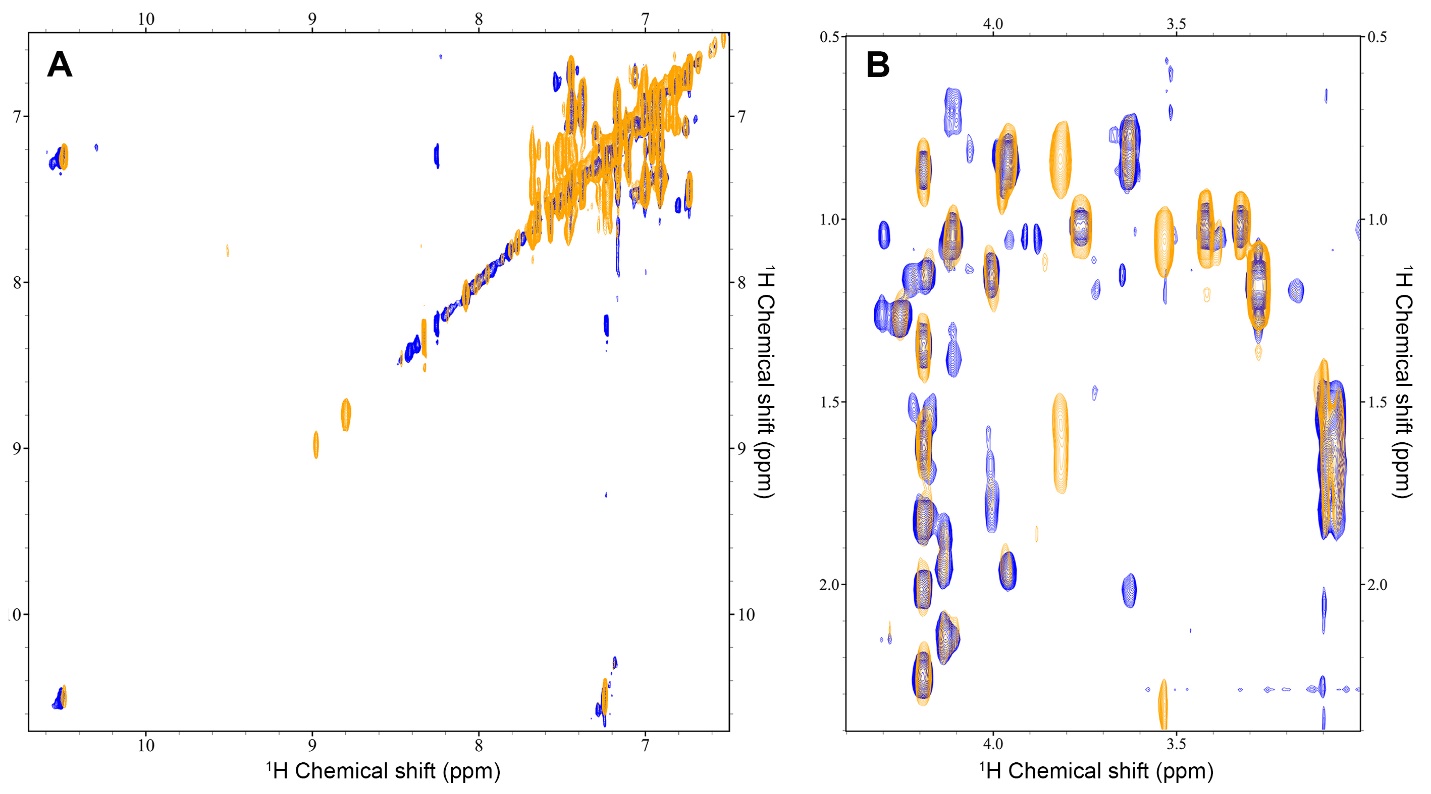
**

**Figure S5.** Aromatic (**A**) and aliphatic (**B**) regions of TOCSY spectra acquired with a 140 ms mixing time for 1 mM sensor peptide solubilised in 100 mM DPC-d_38_, 10 mM DPPC-d_75_, 25 mM sodium phosphate buffer pH 6.8, 25 mM NaCl, 10% D_2_O, in the absence (blue) and presence (orange) of 1 mM vancomycin.


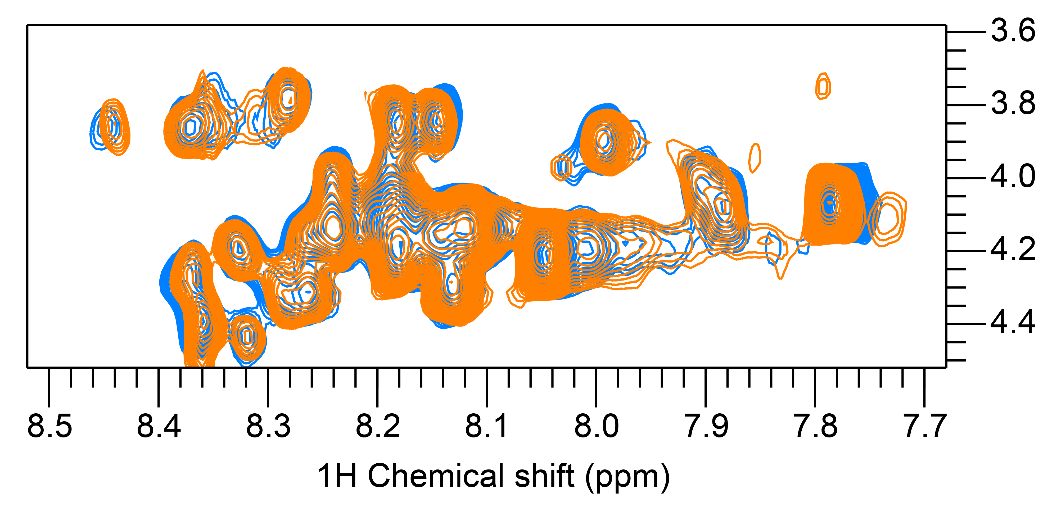


**Figure S6.** The H_N_ - Hα fingerprint region of equivalent TOCSY spectra acquired for 800 μM α-synuclein in DPC/DPPC mixed micelles in the absence (blue) and presence (orange) of 800 μM vancomycin HCl. The H_N_ - Hα crosspeak intensities are unaffected.


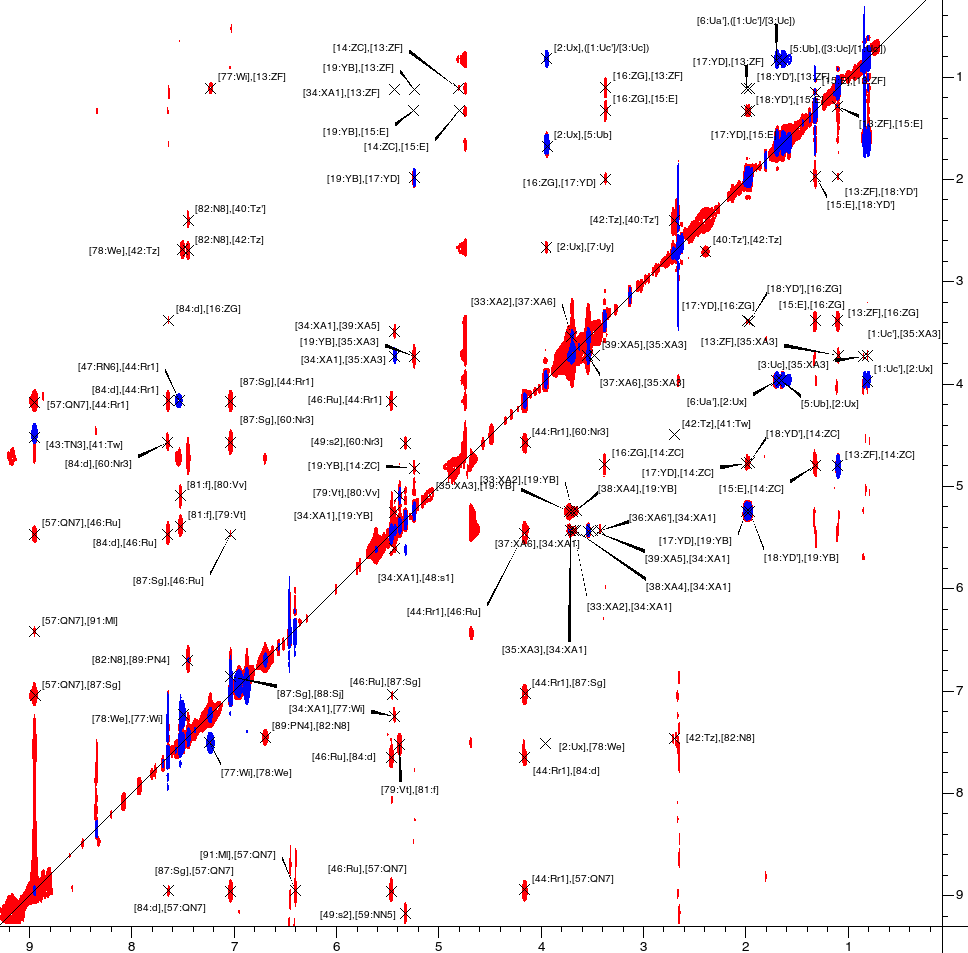


Figure S7. Overlaid 70 ms TOCSY (blue) and 80 ms NOESY (red) spectra of 1 mM vancomycin in 25 mM sodium phosphate buffer pH 6.8, 25 mM NaCl, 10% D_2_O. Assignment was completed in CcpNmr Analysis.

Figure S8. The molecular structure of vancomycin, showing proton chemical shifts in 100 mM DPC-d_38_, 10 mM DPPC-d_75_, 25 mM sodium phosphate buffer pH 6.8, 25 mM NaCl, 10% D_2_O.


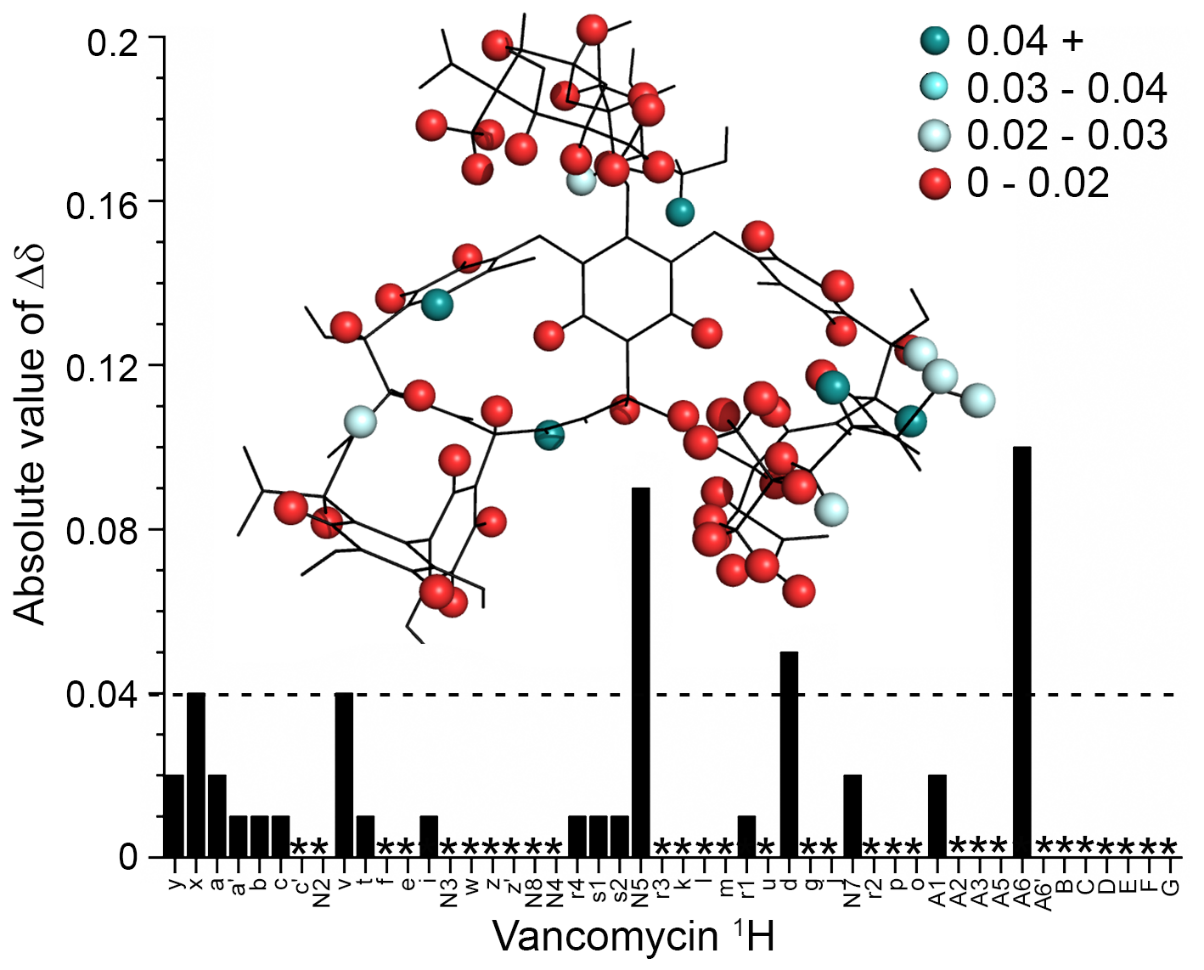


**Figure S9:** The change in chemical shift (Δδ) of each proton in vancomycin HCl upon addition of an equimolar quantity of α-synuclein. Asterisks indicate protons for which no Δδ is observed. The crystal structure of vancomycin (**PDB ID: 1FVM**, crystallised in complex with the lipid II pentapeptide terminus) onto which protons observable in acquired NMR spectra are mapped as solid spheres. The signal perturbations experienced by all observable protons are shown as a heat map on the structure above, with protons experiencing the largest signal perturbation (Δδ ≥ 0.04 ppm, 3 × standard error) shown in dark cyan, and varying in shade between 0.04 - 0.02 ppm. Protons experiencing Δδ from 0 - 0.2 ppm are shown in red to represent negligible perturbation. The presence of α-synuclein causes perturbation of a very few vancomycin protons, which do not cluster together in any discernable binding site.

**
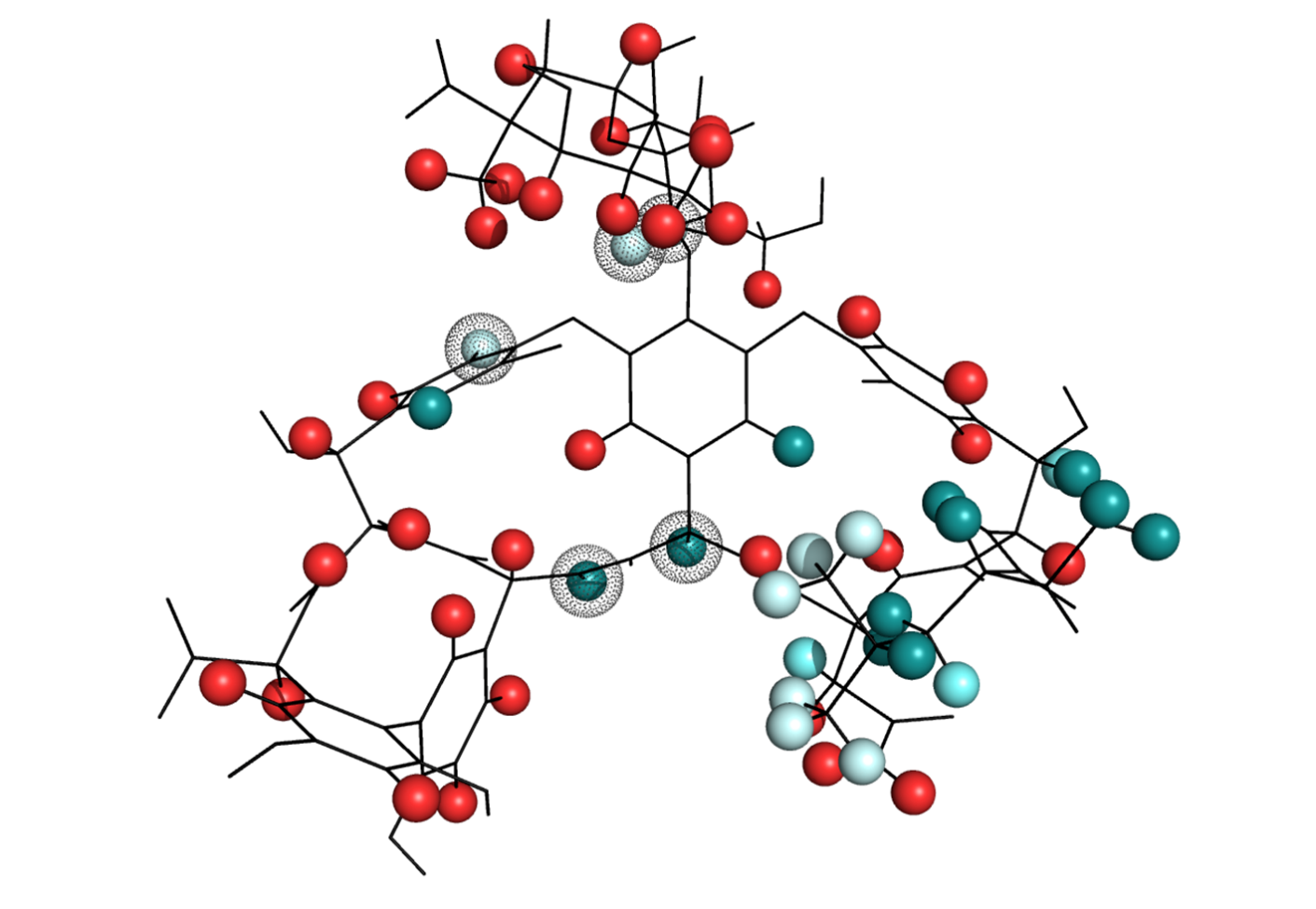
**

**Figure S10:** A vancomycin homodimer is known to assemble above concentrations of 1.4 mM in aqueous solution. Dotted cages indicate protons in each vancomycin monomer which are affected by the presence of the sensor peptide, and localise to the dimerisation interface.

Table S1. ^1^H chemical shift assignments for the sensor peptide solubilized in 100 mM DPC-d_38_, 10 mM DPPC-d_71_, 25 mM sodium phosphate buffer pH 6.8, 25 mM NaCl, 10% D_2_O. Grey fill indicates protons which could not be assigned from the data.

**Table S2.** ^1^H chemical shifts (δ) of 1 mM vancomycin HCl in 100 mM DPC-d_38_, 10 mM DPPC-d_75_, 25 mM sodium phosphate buffer pH 6.8, 25 mM NaCl and 10% D_2_O.

| Proton | ^1^H δ (ppm) | Proton | ^1^H δ (ppm) |
| --- | --- | --- | --- |
| y | 2.67 | l | 6.84 |
| x | 3.99 | m | 6.82 |
| a | 1.56 | r1 | 4.15 |
| a' | 1.78 | u | 5.49 |
| b | 1.63 | d | 7.74 |
| c | 0.90 | g | 7.50 |
| c' | 0.87 | j | 7.23 |
| N2 | 6.95 | N7 | 8.81 |
| v | 4.72 | r2 | 4.47 |
| t | 5.42 | p | 6.39 |
| f | 7.50 | o | 6.50 |
| e | 7.54 | A1 | 5.29 |
| i | 7.25 | A2 | 3.65 |
| N3 | 7.55 | A3 | 3.75 |
| w | 4.12 | A5 | 3.31 |
| z | 2.78 | A6 | 3.72 |
| z' | 2.17 | A6' | 3.59 |
| N8 | 7.58 | B | 5.25 |
| r4 | 5.52 | C | 4.76 |
| s1 | 5.79 | D | 1.99 |
| s2 | 5.26 | E | 1.33 |
| N5 | 8.03 | F | 1.09 |
| r3 | 4.61 | G | 3.39 |
| k | 7.03 |  |  |
